# Rare case of Killian-Pallister syndrome associated with idiopathic short stature detected with fluorescent in situ hybridization on buccal smear

**DOI:** 10.1186/s13039-016-0239-7

**Published:** 2016-05-03

**Authors:** Elena Sukarova-Angelovska, Mirjana Kocova, Gordana Ilieva, Natalija Angelkova, Elena Kochova

**Affiliations:** Department of endocrinology and genetics, University Pediatric Clinic, Medical Faculty, Vodnjanska 17, Skopje, 1000 Macedonia; Genetic laboratory, University Pediatric Clinic, Medical Faculty, Vodnjanska 17, Skopje, 1000 Macedonia; Department of neurology, University Pediatric Clinic, Medical Faculty, Vodnjanska 17, Skopje, 1000 Macedonia; University Pediatric Clinic, Medical Faculty, Vodnjanska 17, Skopje, 1000 Macedonia

**Keywords:** Killian-Pallister syndrome, Isolated growth hormone deficiency, Fluorescent in situ hybridisation, Buccal smear

## Abstract

**Background:**

Killian-Pallister syndrome (KPS) is a rare form of chromosomal mosaicism and is defined by the existence of an extra chromosome 12 in some cell lines in one individual. The degree of mosaicism varies among tissues and dictates the clinical presentation of the syndrome. The clinical features of Killian-Pallister syndrome include mental retardation, typical facial dysmorphism and pigmentation defects.

**Case presentation:**

We present a rare case of Killian-Pallister syndrome with severe form of the disease associated with isolated growth hormone deficiency and low-rate mosaicism on buccal smear. The absence of a marker chromosome 12p in lymphocyte cultures and the low degree of mosaicism lead to frequent misdiagnosis of this condition.

**Conclusions:**

The selection of tissue sampling is crucial in establishing the diagnosis of Killian-Pallister syndrome. Fluorescent in situ hybridisation on buccal smear remains the golden standard as a screening method if a suspicion of the syndrome exists.

## Background

Killian Pallister syndrome (KPS) is a rare chromosomal disorder caused by mosaic tetrasomy of 12p. The syndrome has been described independently by Pallister in 1977 [1], afterwards confirmed by Killian in 1983 [2]. The incidence is estimated to be 1/25,000 [http://www.orpha.net], however these data are still uncertain due to the diagnostic difficulties and unrecognized cases.

In most cases of KPS, the 12p chromosome is present in four copies [3]; 12p trisomy or other complex chromosomal rearrangements involving the short arm of chromosome 12 have rarely been described [4]. The mosaic appearance of isochromosome 12p is tissue-limited and often absent in blood culture. Therefore the diagnosis is frequently delayed until the appearance of clinical features later in life.

The extra chromosome is present in 30–100 % of all analyzed skin fibroblasts and buccal cells, in 10–100 % of bone marrow cells and amniocytes, whereas in lymphocytes the mosaic cell line can be found in 0–2 % [5–7]. Identifying the condition prenatally often fails, since the amniotic cell cultures reveal no chromosomal abnormalities [8].

The clinical presentation is variable, including mild to severe mental retardation, facial dysmorphism, alteration in skin pigmentation (both hypo- and hyperpigmentation) and several nonspecific congenital anomalies. The most significant features that infer the diagnosis are coarse face, full cheeks, sparse hair on the lateral sides of the head, diaphragmal hernias and supernumerary nipples [9]. The presence, severity and combination of the above mentioned features are highly variable, which makes the clinical recognition difficult.

Additionally, certain infrequent and subtle features have been described, such as ear pits, palm vs. finger length, typical upper lip, offering a full dysmorphological spectrum of the syndrome [10, 11]. Epileptic seizures, usually described as generalized or myoclonic, are frequent [12]. Other types of seizures, such as hypsarrhytmia, are rarely described [13]. Alike other syndromic epilepsies, the seizures are hard to control, even with combinations of several antiepileptic drugs.

The growth characteristics in KPS include high birth weight and high growth velocity during the first months, followed by growth retardation and delayed bone age later in childhood. Reynolds [10] suggested that the initial overgrowth typical of KPS needs further elucidation since most of the aneuploidies involving other chromosomes are associated with fetal growth retardation. It is likely that the presence of 3 or 4 copies of certain genes on chromosome 12 provoke fetal overgrowth. Lower birth measurements combined with microcephaly are rarely present [9] and are usually associated with additional anomalies - cardial and intestinal defects, renal cysts, etc.

Some authors suspect that the clinical presentation depends on the degree of mosaicism, while others consider the size of the supernumerary chromosome i.e., the number of present gene copies, more important. However, there are reports of similar degrees of mosaicism but inconsistent severity of clinical features [11]. Some authors suggested that existence of the supernumerary chromosome in particular tissues is responsible for this clinical range rather than degree of mosaicism [14].

## Case presentation

**Fig. 1:**
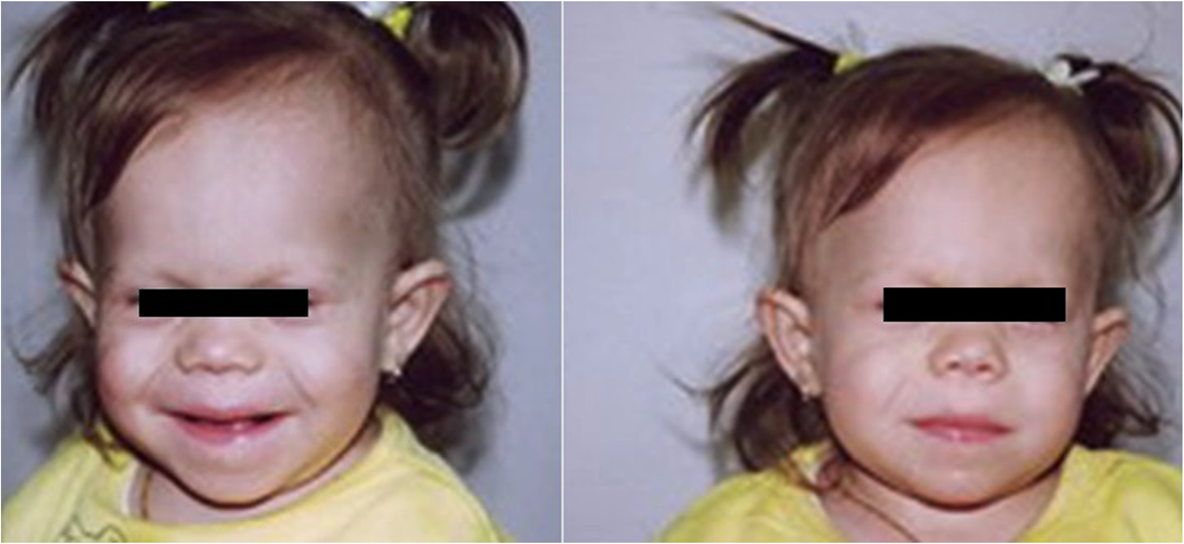
Facial appearance in our patient

The proband is a 14 month-old girl with short stature and mental retardation. This was the first child from healthy unrelated parents. The pregnancy was uneventful; the baby was delivered naturally at 40 weeks of gestation, measuring 2,400 g and 45 cm at birth (small for gestational age). After the delivery generalized hypotonia and a poor sucking reflex were noticed, therefore the baby was fed through a nasogastric tube during the first weeks. During infancy, the growth was continuously under the 3^rd^ percentile; failure to thrive and developmental delay were also noticed. Facial dysmorphic features were present, including: a coarse face, a prominent forehead, bitemporally sparse hair, narrow and upslanted palpebral fissures, hypertelorism, a short and upturned nose with anteverted nares, full cheeks, a long philtrum and protruding lips (Fig. 1).

Standard chromosome analysis of blood lymphocytes was performed, with 3-day cultivation using phytohaemagglutinin and standard harvesting, followed by G-banding [15]. A total of 50 metaphases were analysed using Microscope Olympus BX51 and standard Metasystems karyotyper. The results revealed a normal karyotype in all analysed cells.

**Fig. 2:**
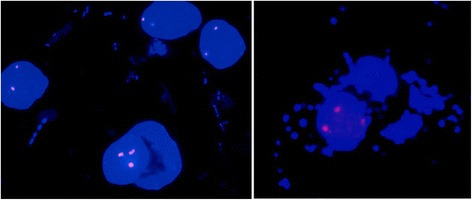
FISH for centromeric probe of chromosome 12 performed on buccal smear, showing 3 signals in 30 % of the analyzed cells

Buccal smear was dissolved in 2 ml 1xPBS solution, centrifuged and put into fixative (3:1 acetic acid-methanol solution). After centrifugation and supernatant disposal, solution of 0,075 M KCL on 37 °C temperature was added to the sediment. The fixing procedure was repeated two times successively; afterwards the sediment was transferred on the slide. The dried slide was placed in solution of 0,001 N HCL and 100 μl pepsin for 10 min in water tub on 37 °C. Fluorescent in situ hybridization (FISH) was performed using the centromeric probe (red signal) for chromosome 12 (12p, Vysis, CEP 12 spectrum orange, cat. No 30–160012). The standard FISH protocol was used [16]. The fluorescent light microscope analysis (Olympus BX51) was performed both on blood leukocytes and buccal smear. Adjacent filters were used – 4’,6-diamidino-2-phenylindole (DAPI) for the counterstaining, and tetramethylrhodamine isothiocyanate (TRITC) for the visualization of red colour. Computer analysis was done with Metasystems-FISH software, version 5.5. At least 200 nuclei from each tissue were analysed. The extra 12p signal was detected in 30 % of the analysed buccal smear cells, but not in the blood leukocyte culture (Fig. 2). The fibroblast culture was not performed due to the parental denial and condition of the patient.

**Fig. 3:**
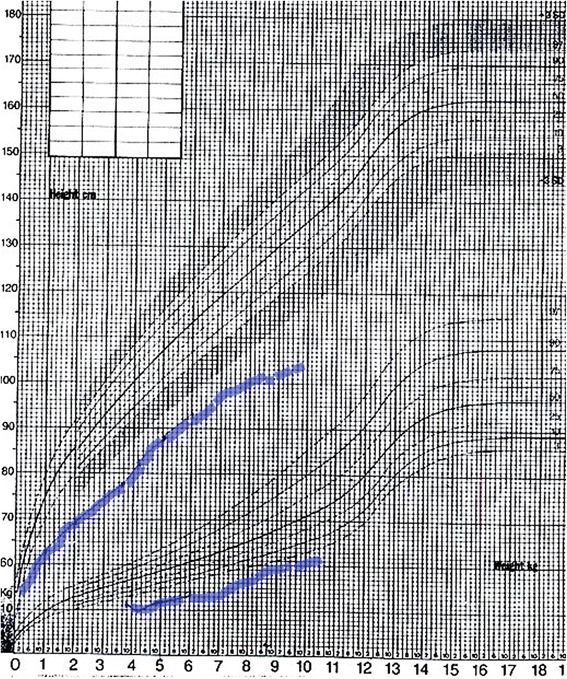
Growth curve representing prenatal and postnatal growth retardation

**Fig. 4:**
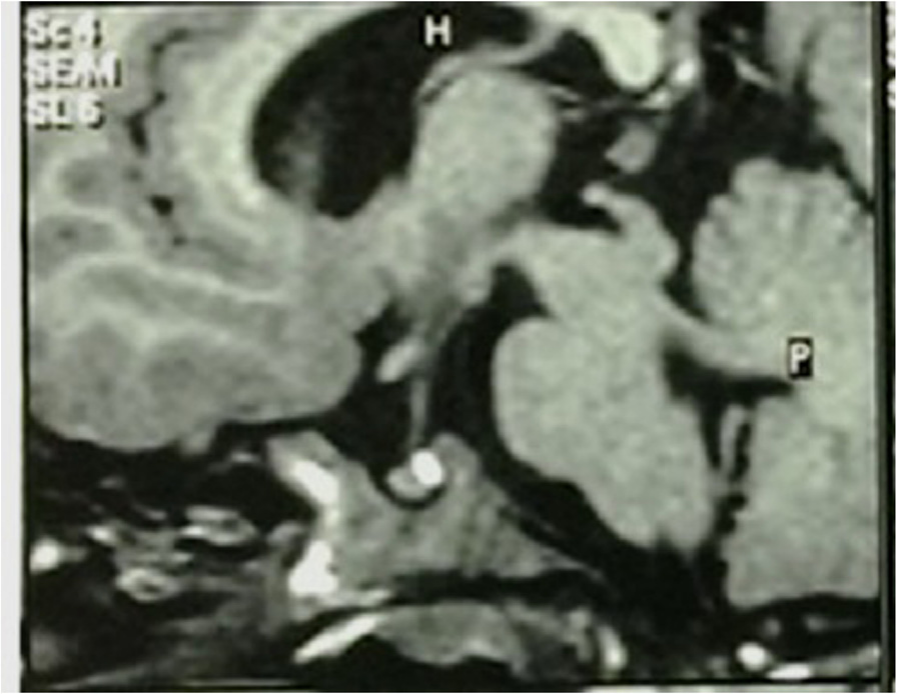
MRI of the brain showing slightly enlarged ventricles and pituitary gland with no abnormalities

At the age of 2.5 years the short stature became more evident - it was 3.5 SDS below the mean (Fig. 3). GH stimulation tests were performed, with a peak value of 4.9 ng/ml; insulin-like growth factor 1 (IGF1) was also below the expected values - under 10 ng/ml (ref. value 24–152 ng/ml for the age). MRI of the pituitary showed normal structure (Fig. 4). The other pituitary hormone levels were normal.

**Fig. 5:**
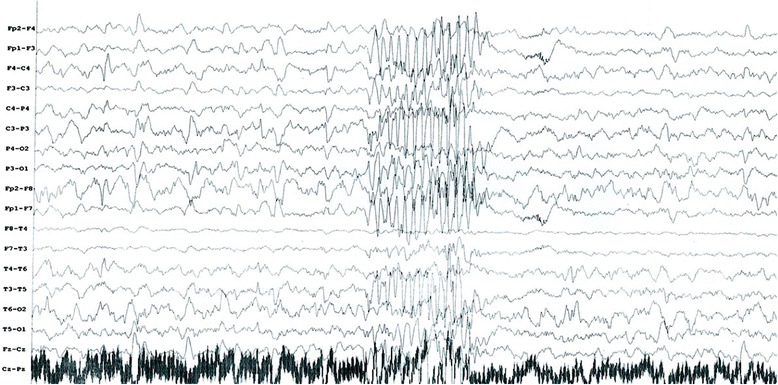
Right-sided spike-and-wave focus in the fronto-temporo-parietal region, with a short generalized discharge on EEG

At the age of 4 years the child developed clonic-tonic seizures and her motor and mental abilities started to deteriorate - she lost some of her previously acquired achievements. She developed profound mental retardation and incontinence. The seizures were resistant to anticonvulsive therapy (Fig. 5). Due to the presence of intractable epilepsy and mental deterioration, GH replacement therapy was not initiated. Puberty had an early onset - at the age of 8.5 years, leading to further growth delay.

## Discussion

Establishing the diagnosis of Killian-Pallister syndrome (KPS) is often difficult and demanding, requiring thorough clinical observation and different cytogenetic and molecular methods. The underlying mechanism for this rare chromosomal error is prezygotic [17], occurring in maternal meiosis 2 followed by mitotic loss of the marker chromosome in some cells. The predominance of the meiosis 2 error in the formation of i(12p), unlike other chromosomopathies, is still unresolved. Dufke [18] clarified the smallest critical region of the chromosome (12pter-12p12.3) using different molecular-cytogenetic techniques. The phenotypic characteristics of KPS were confirmed to be a result of the dosage effect of LDH-B gene in some cases [19], but others indicate that a fitting phenotype is possible without the presence of this gene [18]. In all cases, however, heterozygosity is reduced to homozigosity in the derivative chromosome 12. Kaur [20] gives a comprehensive analysis chromosome 12 genes that could produce a KPS phenotype, narrowing the critical region on band 12p13.31. He identified several genes that, when misexpressed (either up or down regulated), yield clinical features of the syndrome.

The growth curve in KPS patients is biphasic - prenatal overgrowth followed by postnatal growth retardation. Although there are several genes on chromosome 12 that promote growth in various tissues (ADIPOR2, FGF6, NTF3, KRAS1, etc.), there are no specific studies about the reason for fetal overgrowth in these patients. Unlike other children with KPS, in our patient growth retardation was present both prenatally (the baby was born small for gestational age) and postnatally (constantly 3SDS below the mean). Growth hormone deficiency is an uncommon finding in KPS, described only once in the literature [21] and should be further evaluated. Overexpression of insulin-like growth factor 2 (IGFBP2) is a probable reason for postnatal growth retardation [20]. The reason for prenatal growth retardation in our case is unclear.

The cytogenetic finding of i(12p) is rarely detected in blood leukocytes. Some speculate that this is due to the distortion of abnormal cells during the culturing procedure, during which i(12p) becomes dramatically reduced [3, 22]. This reduction occurs in vivo as well; therefore the aneuploidy can be detected in bone marrow tissue only in the early neonatal period [23]. In all other tissues that divide less frequently (i.e.fibroblasts) the extra chromosome persists for longer periods and can be detected later in life. Therefore skin biopsy is most reliable method in detecting the syndrome.

The use of the FISH method in tissues other than blood leukocytes was first described by Speleman [24] who detected i(12p) in fibroblasts; the value of the method was afterwards confirmed by other authors [25, 26] who used FISH on buccal smear. The method allows detection of i(12p) on interphase nuclei without cultivation and is considered to be a reliable, rapid and effective method for the detection of the chromosomal abnormality in this syndrome. The presence of low-rate mosaicism of i(12p) broadens the phenotypic spectrum of the syndrome. In most of the studies so far [25, 27], an alpha satellite probe of the centromeric region of chromosome 12 was used, but some authors [26, 27] suggest the usage of a dual colour, locus specific probe in order to avoid cross-hybridisation with other cetromeres and false positive results, as well as to detect the number and length of the supernumerary 12p. Recently, other methods such as array CGH are proposed as more sensitive [14, 22]; however these methods are more expensive, require more sophisticated equipment and could not be used as a screening procedure. Yet essential for diagnosis is not the method used, but the adequate choice of tissue for sampling.

## Conclusions

The FISH method on buccal smear remains invaluable for the detection of Killian-Pallister syndrome. In order to avoid diagnostic delay and invasive diagnostic procedures, the buccal smear FISH analysis remains a superior method due to the less invasive nature of sample collection.

Underlying mechanisms of the biphasic growth pattern in Killian Pallister syndrome are not yet elucidated and need further investigations. Rarely, both prenatal and postnatal growth pattern are affected and could be associated with growth hormone deficiency, as shown in our case.

### Consent

"Written informed consent was obtained from the patient for publication of this case report and any accompanying images. A copy of the written consent is available for review by the Editor-in-Chief of this journal."
